# INDIGENA: inductive prediction of disease–gene associations using phenotype ontologies

**DOI:** 10.1093/bioinformatics/btag325

**Published:** 2026-05-21

**Authors:** Fernando Zhapa-Camacho, Robert Hoehndorf

**Affiliations:** Computer, Electrical and Mathematical Sciences & Engineering Division, King Abdullah University of Science and Technology, Thuwal 23955, Saudi Arabia; KAUST Center of Excellence for Smart Health (KCSH), King Abdullah University of Science and Technology, Thuwal 23955, Saudi Arabia; KAUST Center of Excellence for Generative AI, King Abdullah University of Science and Technology, Thuwal 23955, Saudi Arabia; Computer, Electrical and Mathematical Sciences & Engineering Division, King Abdullah University of Science and Technology, Thuwal 23955, Saudi Arabia; KAUST Center of Excellence for Smart Health (KCSH), King Abdullah University of Science and Technology, Thuwal 23955, Saudi Arabia; KAUST Center of Excellence for Generative AI, King Abdullah University of Science and Technology, Thuwal 23955, Saudi Arabia

## Abstract

**Motivation:**

Predicting gene–disease associations (GDAs) can be framed as a ranking problem where genes are ranked for a query disease based on features such as phenotypic similarity. By describing phenotypes using phenotype ontologies, ontology-based semantic similarity measures can be used. However, traditional semantic similarity measures use only the ontology taxonomy. Recent methods based on ontology embeddings compare phenotypes in latent space; these methods can use all ontology axioms as well as a supervised signal, but are inherently transductive, i.e. query entities must already be known at the time of learning embeddings, and therefore these methods do not generalize to novel diseases (sets of phenotypes) at inference time.

**Results:**

We developed INDIGENA, an **in**ductive **di**sease–**gen**e **a**ssociation method for ranking genes based on a set of phenotypes. Our method first uses a graph projection to map axioms from phenotype ontologies to a graph structure, and then uses graph embeddings to create latent representations of phenotypes. We use an explicit aggregation strategy to combine phenotype embeddings into representations of genes or diseases, allowing us to generalize to novel sets of phenotypes. We also develop a method to make the phenotype embeddings and the similarity measure task-specific by including a supervised signal from known GDAs. We apply our method to mouse models of human disease and demonstrate that we can significantly improve over the inductive semantic similarity baseline measures, and reach a performance similar to transductive methods for predicting GDAs while being more general.

**Availability and implementation:**

https://github.com/bio-ontology-research-group/indigena.

## 1 Introduction

Gene–disease associations (GDAs) for Mendelian diseases can be identified through multiple computational approaches. Mendelian diseases, characterized by single-gene mutations following Mendelian inheritance patterns, are predominantly rare disorders affecting fewer than one in 2000 individuals (Umair and Waqas 2023). Computational methods for identifying these associations include: (i) guilt-by-association methods that leverage biological networks where genes with similar network properties to known disease genes are implicated in similar diseases (Köhler *et al.* 2008), (ii) phenotype similarity to databases of patients or diseases that connects patients with similar phenotypic profiles to known genetic causes (Köhler *et al.* 2009), and (iii) phenotype similarity to model organisms, which provides substantially more data for inference (Hoehndorf *et al.* 2011). This last approach is particularly useful as wet lab validation of GDAs remains time-consuming and expensive (Nunes 2021), while computational methods can efficiently leverage large gene–phenotype and disease–phenotype datasets (Yuan *et al.* 2022). For rare Mendelian diseases, these approaches are crucial for advancing diagnosis and treatment options for affected patients.

Phenotypes are recorded using standardized ontologies that enable computational analysis. Human phenotypes are described using the human phenotype ontology (HPO) (Gargano *et al.* 2024), while mouse phenotypes are captured in the mammalian phenotype (MP) ontology (Smith and Eppig 2009). These species-specific ontologies structure phenotypes hierarchically with formal logical definitions. Cross-species ontologies such as UPheno (Matentzoglu *et al.* 2024) and PhenomeNET (Hoehndorf *et al.* 2011) facilitate comparisons between human and mouse phenotypes by relating classes of phenotypes in different species axiomatically and thereby making them comparable.

Phenotypes are then used to predict GDAs through a ranked retrieval approach. This process involves using a phenotype similarity measure (a semantic similarity measure) to query a database of genotype–phenotype associations, e.g. from the Online Mendelian Inheritance in Man (OMIM) (Amberger *et al.* 2015) or the mouse genome informatics (MGI) (Baldarelli *et al.* 2024) databases. Genotypes, usually representing loss of function of one or two alleles of a gene, are then ranked based on their phenotype similarity to the query disease. This ranking enables prioritization of candidate genes that are most likely to be causally related to a disease (Gkoutos *et al.* 2018). The effectiveness of this approach relies on the accuracy of the phenotype similarity measure and how complete the underlying phenotype data is.

Traditional semantic similarity measures for phenotype comparison are typically handcrafted (their formulas are designed by domain experts rather than learned from data) and are inductive (can generalize to novel phenotype sets for querying) (Harispe *et al.* 2015). Examples include Resnik’s information content (IC)-based measure (Resnik 1995) combined with the best match average (BMA) approach (Pesquita *et al.* 2009) for combining multiple comparisons.

These measures have been successfully applied to the GDA prediction task (Hoehndorf *et al.* 2011, Chen *et al.* 2012, Smedley *et al.* 2013, Alghamdi *et al.* 2022, Putman *et al.* 2024). However, semantic similarity measures primarily rely on the phenotype ontology’s hierarchical structure and do not consider other axioms between phenotypes. Furthermore, because semantic similarity measures are handcrafted, they do not adapt to the data or task of predicting GDAs. More recent work has applied machine learning to generate embeddings of phenotypes, genes, and diseases. These knowledge graph or ontology embeddings (Chen *et al.* 2025) learn latent representations of single phenotypes or sets of phenotypes, which can then be used either through a vector similarity measure to perform ranked retrieval, or using a supervised method like a learning-to-rank approach with a neural network (Chen *et al.* 2021b). Supervised embedding-based methods applied to the task of predicting GDAs based on phenotype similarity include Onto2Vec (Smaili *et al.* 2018) and OPA2Vec (Smaili *et al.* 2019), DL2Vec (Chen *et al.* 2021b), OWL2Vec* (Chen *et al.* 2021a), and SmuDGE (Alshahrani and Hoehndorf 2018).

Embedding-based approaches are inherently transductive, which requires that all entities (diseases, genes) that will be used during inference must already be available during the training phase. This means that these models cannot generalize to previously unseen diseases without complete retraining, thereby limiting their applicability to patients with a previously known disease. Furthermore, the embedding methods that were applied to the task of predicting GDAs only actually improve predictive performance over traditional semantic similarity measures when they incorporate a supervised signal, known GDAs, during training. This supervised signal may introduce bias as the model can “memorize” known associations rather than learning generalizable patterns from phenotype data alone (Alghamdi *et al.* 2022), or predict entirely based on the number of times a certain disease or gene was seen during training (Smaili *et al.* 2019).

The limitations of transductive approaches extend beyond basic GDA prediction to variant prioritization applications. Systems such as Exomiser (Robinson *et al.* 2014) or EmbedPVP (Althagafi *et al.* 2024) combine phenotype-based GDA prediction with variant pathogenicity measures to prioritize potentially causal variants in clinical settings. For such applications, the ability to make inductive predictions is even more critical. Patients with rare or previously uncharacterized genetic conditions cannot benefit from approaches that require prior knowledge of their specific disease during model training. While systems like Exomiser use semantic similarity, recent embedding-based methods like EmbedPVP (Althagafi *et al.* 2024) use a transductive method for predicting GDAs; therefore, while they show a higher predictive performance than methods based on semantic similarity, they are more limited in their application. On the other hand, attempts to extend embedding-based methods to the inductive setting (Althagafi *et al.* 2024, Bakheet 2025) resulted in predictive performance that did not reach or exceed that of classical semantic similarity measures.

We developed INDIGENA, a fully **in**ductive method for **di**sease–**gen**e **a**ssociation prediction based on ontology embeddings while retaining a supervised learning component. Our approach enables ranking genes based on phenotype similarity without requiring the test diseases (sets of phenotypes) to be present during training. Building on the early proof of concept explored in Bakheet (2025), we provide a complete implementation, a systematic evaluation across multiple embedding methods and graph structures, and a new analysis of why projection-based models are particularly suited for this task. We find that INDIGENA outperforms traditional semantic similarity measures while maintaining the ability to generalize to previously unseen diseases.

Our main contributions include: (i) an inductive framework for GDA prediction that generalizes to new combinations of phenotypes; (ii) an empirical validation of our method’s effectiveness compared to established semantic similarity measures; and (iii) a systematic analysis of how different graph structures and embedding methods affect inductive prediction performance. We make our code available as Free Software at https://github.com/bio-ontology-research-group/indigena.

## 2 Materials and methods

### 2.1 Datasets

We obtained gene–phenotype associations from the MGI database (Baldarelli *et al.* 2024). Specifically, we used the file MGI_GenePheno.rpt, downloaded from the Mouse Genome Informatics Database on 20 August 2025. From this file we extracted gene identifiers and their corresponding phenotypes, encoded with the MP.

For disease–phenotype associations, we used the file phenotype.hpoa from the HPO database (Gargano *et al.* 2024), downloaded on 20 August 2025.

We used the UPheno cross-species phenotype ontology (Matentzoglu *et al.* 2024), downloaded from Github (https://github.com/obophenotype/upheno-dev/releases/tag/v2025-07-21) with release date 21 July 2025. For all phenotype associations, we ensured that the phenotypes exist in the UPheno ontology, otherwise we omit the phenotype association.

To evaluate our ability to identify GDAs, we used the file MGI_Geno_DiseaseDO.rpt from the Mouse Genome Informatics Database (Baldarelli *et al.* 2024), downloaded on 20 July 2025.

### 2.2 Ontology preprocessing and graph projection

**Figure 1:**
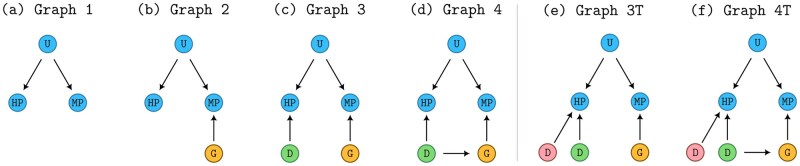
Different graph structures. Nodes 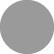 represent UPheno entities, 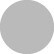 represent genes, 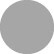 represent training diseases, and 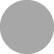 represent testing diseases. (a) Graph 1 is the original UPheno graph representation. (b) Graph 2 includes gene–phenotype associations. (c) Graph 3 includes disease–phenotype associations. (d) Graph 4 includes gene–disease associations. (e) and (f) Transductive variations where testing diseases have been added to the graph linking them to their phenotypes.

We define an ontology as the tuple O=(Σ,Ax), where Σ=(C,R,I) provides the signature of the ontology (C is a set of class names, R is a set of role names, and I is a set of individual names) and *Ax* provides the set of axioms over Σ. We use the axioms in the UPheno ontology and added new axioms representing gene–phenotype, disease–phenotype, and GDAs. For example, for an association between a gene $g_{i}$ and phenotype $p_{j}$, we created the axiom $g_{i}\;⊑\exists \;\text{has}\_\text{phenotype}.p_{j}$. Similarly, disease–phenotype associations were transformed to axioms $d_{i}\;⊑\exists \;\text{has}\_\text{symptom}.p_{j}$ and disease–gene associations as axioms $d_{i}\;⊑\exists \;\text{associated}\_\text{with}.g_{j}$. A graph projection maps O into a graph G following a specific set of rules (Zhapa-Camacho and Hoehndorf 2023). We projected UPheno and its extensions into graphs following the projection rules designed by OWL2Vec* (Chen *et al.* 2021a). To evaluate the impact of different information sources on prediction performance, we constructed four distinct datasets with increasing amounts of information:

- Graph 1 contains only the UPheno ontology, with its phenotype names for humans and mice. This baseline graph includes the hierarchical relationships between phenotype terms and the cross-species mappings between human and mouse phenotypes, but no gene or disease annotations (Fig. 1a).
- Graph 2 extends Graph 1 with gene–phenotype associations, connecting MGI gene identifiers to their associated MP terms. The phenotypes associated with a gene are those observed when the mouse genes are mutated. Note that the set of genes is always known and does not change (Fig. 1b).
- Graph 3 extends Graph 2 with disease–phenotype associations, connecting OMIM disease identifiers to their associated HPO phenotype terms. This graph contains the complete phenotypic profiles of both genes and diseases. As long as a testing disease (or set of phenotypes) is not included in this graph, predicting GDAs with Graph 3 is inductive (Fig. 1c).
- Graph 4 extends Graph 3 with known GDAs between MGI genes and OMIM diseases (i.e. mouse models of human disease). This graph contains the complete information set, including the ground truth associations that we used for ontology embedding learning (Fig. 1d).

Graphs 3T and 4T (Fig. 1e and f) are transductive variations, where test diseases are added as nodes to the training graph with their phenotype associations only (not the GDAs).

These four graph structures allow us to systematically evaluate how additional information affects the model’s ability to predict GDAs. By comparing performance across these structures, we can determine whether the inclusion of gene–phenotype, disease–phenotype, or direct GDAs impacts prediction accuracy.

To ensure our method is truly inductive and can generalize to previously unseen diseases, we implemented a 10-fold cross-validation strategy based on disease splits. For Graphs 3 and 4, which contain disease entities, the disease set was randomly partitioned into 10 equally-sized subsets. In each fold, 90% of diseases were used for training together with their gene associations and 10% were held out for testing. Additionally, we remove diseases from the testing set when such diseases have the same phenotypic profile and the same gene associations as any disease in the training set. This procedure allows training on known GDAs while ensuring that the diseases (or their phenotype set) used for evaluation were never seen during the training phase. This setup validates the model’s ability to make predictions on novel diseases based solely on their phenotypic profiles.

### 2.3 Graph embedding methods

We note that the term “projection” is used in two distinct senses in this work. The *graph projection* described in Section 2.2 is a preprocessing step that maps an OWL ontology into a labeled graph by applying syntactic rewriting rules to its axioms (following OWL2Vec*). In contrast, the *entity projection* used by some knowledge graph embedding models (e.g. TransH and TransD) is a learned linear map that projects entity embeddings into a relation-specific subspace before the scoring function is applied. The first operates on axioms to produce a graph; the second operates on vectors to produce relation-specific representations.

We use several knowledge graph embedding methods (Wang *et al.* 2017) for our experiments, specifically TransE (Bordes *et al.* 2013), TransH (Wang *et al.* 2014), TransD (Ji *et al*. 2015) and ConvKB (Nguyen *et al.* 2018). Each embedding method captures different entity and relation patterns from the knowledge graph into the embedding space depending on the scoring function f(h,r,t) (Table 1). TransE models relationships as translations in the embedding space. TransH projects entities into relation-specific hyperplanes, where relations are interpreted as translations. TransD introduces entity-specific projection vectors that allow projecting head and tail entities differently. While the Trans{E, H, D} methods provide a geometric interpretation of embeddings, we also use ConvKB, which employs a convolutional neural network on the concatenated embeddings of entities and relations. The original ConvKB model is initialized with TransE embeddings. Furthermore, we explore the impact of initializing ConvKB with TransD embeddings, which we name ConvKB-D. For each method, we tune the following hyperparameters: embedding dimension [100, 200, 300, 400], batch size [1024, 2048, 4096, 8192], learning rate [0.0001, 0.001, 0.01], and number of convolutional filters (only for ConvKB and ConvKB-D) [100, 200]. The selected list of hyperparameters is shown in Table 1, available as supplementary data at *Bioinformatics* online.

**Table 1:** Summary of knowledge graph embedding models.

Method	Ent. embedding	Rel. embedding	Scoring function
TransE	$h,t\in R^{d}$	$r\in R^{d}$	$f(h,r,t)=-||h+r-t|{|}_{1/2}$
TransH	$h,t\in R^{d}$	$r,w_{r}\in R^{d}$	$f(h,r,t)=-||(h-w_{r}^{⊤}hw_{t})+r-(t-w_{r}^{⊤}tw_{r})|{|}_{2}^{2}$
TransD	$h,t,w_{h},w_{t}\in R^{d}$	$r,w_{r}\in R^{k}$	$f(h,r,t)=-||(w_{r}w_{h}^{⊤}+I)h+r-(w_{r}w_{t}^{⊤}+I)t|{|}_{2}^{2}$
ConvKB	$h,t\in R^{d}$	$r\in R^{d}$	$f(h,r,t)=[v_{1};\ldots ;v_{\tau }]\cdot w,\;v_{i}=g(\omega_{j}[h,r,t]+b),\;w\in R^{\tau d}$

### 2.4 Semantic similarity

Semantic similarity measures quantify the likeness of concepts based on their meaning and relationships within an ontology (Pesquita *et al.* 2009). For phenotype-based GDA prediction, we use two approaches: semantic similarity measures and embedding-based similarity. Regarding semantic similarity measures we used Resnik (1995) and Lin (1998). These measures quantify the similarity between two terms in an ontology based on their shared IC with Lin’s measure being a variation of Resnik’s. For two phenotype terms $p_{1}$ and $p_{2}$, the similarity is defined as:


(1) $$sim_{\mathrm{Resnik}}(p_{1},p_{2})=IC(\mathrm{MICA}(p_{1},p_{2}))$$

(2) $$sim_{\mathrm{Lin}}(p_{1},p_{2})=\frac{2\cdot IC(\mathrm{MICA}(p_{1},p_{2}))}{IC(p_{1})+IC(p_{2})}$$

where $\mathrm{MICA}(p_{1},p_{2})$ is the most informative common ancestor of $p_{1}$ and $p_{2}$ in the ontology hierarchy, and IC(p) is the IC of term *p*, calculated as:


(3) IC(p)=−log(P(p))

with P(p) representing the frequency of phenotype term *p* in the corpus of annotations. We used the semantic measures library (Harispe *et al.* 2014, 2015) to compute semantic similarity measures.

To compute similarity between sets of terms, we rely on two indirect approaches (i.e. measures that first compute pairwise measures and then combine), the BMA and best match maximum (BMM), and one direct approach (i.e. a measure that does not rely on pairwise comparisons), SimGIC (Pesquita *et al.* 2007):


(4) $$sim_{\mathrm{BMA}}(P_{g},P_{d})=\frac{1}{2}(\frac{\sum_{i=1}^{n}\max_{j}sim(p_{gi},p_{dj})}{n}+\frac{\sum_{j=1}^{m}\max_{i}sim(p_{gi},p_{dj})}{m})\;$$

(5) $$sim_{\mathrm{BMM}}(P_{g},P_{d})=\max(\frac{\sum_{i=1}^{n}\max_{j}sim(p_{gi},p_{dj})}{n},\frac{\sum_{j=1}^{m}\max_{i}sim(p_{gi},p_{dj})}{m})$$

(6) $$sim_{\mathrm{GIC}}(P_{g},P_{d})=\frac{\sum_{p\in C_{T}^{+}(P_{g})\cap C_{T}^{+}(P_{d})}^{n}IC(c)}{\sum_{p\in C_{T}^{+}(P_{g})\cup C_{T}^{+}(P_{d})}^{n}IC(c)}$$

where $C_{T}^{+}(X)$ is the union of ancestors of the concepts in *X*.

### 2.5 Embedding-based similarity

To provide an inductive prediction framework, we use the vector representations generated by knowledge graph embedding models. Notice that graph embedding methods are optimized with the scoring function f(h,r,t). However, we work under the assumption that the embeddings capture similarity features during the training process. Therefore, given two entity terms with embeddings $\vec{p_{1}}$ and $\vec{p_{2}}$, we calculate their similarity as follows:


(7) $$sim^{e}(p_{1},p_{2})=\sigma (\langle \vec{p_{1}},\vec{p_{2}}\rangle )$$

where 〈·,·〉 represents the dot product between vectors and σ(·) is the sigmoid function. For a gene *g* and disease *d*, we can compute their similarity in two ways: (a) using the entities directly in the expression $sim^{e}(g,d)$ or (b) using their phenotype representations with different aggregation methods such as BMA (Equation (8)) and BMM (Equation (9))


(8) $$sim_{\mathrm{BMA}}^{e}(P_{g},P_{d})=\frac{1}{2}(\frac{\sum_{i=1}^{n}\max_{j}sim^{e}(p_{gi},p_{dj})}{n}+\frac{\sum_{j=1}^{m}\max_{i}sim^{e}(p_{gi},p_{dj})}{m})$$

(9) $$sim_{\mathrm{BMM}}^{e}(P_{g},P_{d})=\max(\frac{\sum_{i=1}^{n}\max_{j}sim^{e}(p_{gi},p_{dj})}{n},\frac{\sum_{j=1}^{m}\max_{i}sim^{e}(p_{gi},p_{dj})}{m})$$

### 2.6 Evaluation metrics

We report standard ranking-based metrics. For each test disease, all candidate genes are ranked by the scoring function and the rank of each true causative gene is recorded. From these ranks we compute: the mean rank (MR, the average rank of true associations; lower is better), the mean reciprocal rank (MRR, the average of 1/rank; higher is better), Hits@*k* (H@*k*, the fraction of true associations ranked within the top *k*; higher is better), and the area under the ROC curve (AUC, the probability that a true association is ranked above a random non-association; higher is better).

## 3 Results

### 3.1 Transductive prediction of GDAs

We start analysing the prediction capabilities of embedding methods in a transductive setting. For this task, we use Graphs 3T and 4T, which contain the testing diseases in the training set. Graph 4T also includes GDAs for training diseases; therefore we say Graph 4T includes a *supervised signal*.

We evaluated five different knowledge graph embedding methods: TransE, TransH, TransD, ConvKB, and ConvKB-D, and show the results in Table 2. First, we find that introducing a supervised signal (Graph 4T) generally performs better than using no supervised information (Graph 3T). While there are some exceptions (TransE), most embedding methods can take advantage of the supervised signal. Second, embeddings can capture similarity features during training.

**Table 2:** Performance comparison of different gene–disease association prediction methods.^a^

Method	Scoring	**MR (** ↓ **)**	**MRR (** ↑ **)**	**H@100 (** ↑ **)**	**AUC (** ↑ **)**	**MR (** ↓ **)**	**MRR (** ↑ **)**	**H@100 (** ↑ **)**	**AUC (** ↑ **)**
			
		Non-supervised signal (Graph 3T)				Supervised signal (Graph 4T)			
SimGIC	$sim_{\mathrm{GIC}}$	274.92±31.21	0.08±0.02	0.50±0.05	0.82±0.02	274.92±31.21	0.08±0.02	0.50±0.05	0.82±0.02
Resnik	$sim_{\mathrm{BMM}}$	221.53±26.04	0.08±0.01	0.51±0.06	0.86±0.02	221.53±26.04	0.08±0.01	0.51±0.06	0.86±0.02
Resnik	$sim_{\mathrm{BMA}}$	176.01±18.07	**0.12** ± 0.02	0.62±0.04	0.89±0.01	176.01±18.07	**0.12** ± 0.02	0.62±0.04	0.89±0.01
Lin	$sim_{\mathrm{BMM}}$	209.27±16.12	0.08±0.01	0.52±0.03	0.87±0.01	209.27±16.12	0.08±0.01	0.52±0.03	0.87±0.01
Lin	$sim_{\mathrm{BMA}}$	164.67±13.68	**0.12** ± 0.01	0.64±0.03	0.89±0.01	164.67±13.68	**0.12** ± 0.01	0.64±0.03	0.89±0.01
TransE	f(h,r,t)	–	–	–	–	266.45±27.53	0.06±0.01	0.45±0.03	0.83±0.02
	$sim^{e}(h,t)$	273.42±12.65	0.04±0.01	0.41±0.04	0.82±0.01	318.51±27.93	0.04±0.01	0.36±0.05	0.79±0.02
	$sim_{\mathrm{BMM}}^{e}$	412.15±29.01	0.02±0.01	0.25±0.03	0.73±0.02	426.90±28.14	0.02±0.01	0.22±0.05	0.72±0.02
	$sim_{\mathrm{BMA}}^{e}$	355.27±22.30	0.04±0.01	0.33±0.04	0.77±0.01	367.84±24.38	0.03±0.01	0.30±0.05	0.76±0.02
TransH	f(h,r,t)	–	–	–	–	575.77±36.97	0.01±0.00	0.13±0.03	0.63±0.02
	$sim^{e}(h,t)$	548.78±33.73	0.01±0.00	0.16±0.04	0.64±0.02	458.51±44.40	0.02±0.01	0.23±0.04	0.70±0.03
	$sim_{\mathrm{BMM}}^{e}$	349.73±42.41	0.04±0.01	0.34±0.04	0.77±0.03	301.94±35.05	0.04±0.02	0.39±0.06	0.80±0.02
	$sim_{\mathrm{BMA}}^{e}$	299.72±50.42	0.05±0.02	0.43±0.06	0.81±0.03	247.87±29.34	0.06±0.02	0.47±0.06	0.84±0.02
TransD	f(h,r,t)	–	–	–	–	311.19±54.84	0.06±0.02	0.41±0.07	0.80±0.04
	$sim^{e}(h,t)$	273.69±53.55	0.07±0.01	0.48±0.05	0.82±0.03	238.20±53.44	0.07±0.02	0.50±0.08	0.85±0.03
	$sim_{\mathrm{BMM}}^{e}$	180.35±36.59	0.08±0.01	0.58±0.06	0.88±0.02	164.51±28.79	0.09±0.02	0.63±0.05	0.89±0.02
	$sim_{\mathrm{BMA}}^{e}$	148.32±33.70	0.11±0.02	**0.66** ± 0.06	**0.91** ± 0.02	134.66±25.69	**0.12** ± 0.02	**0.70** ± 0.05	**0.91** ± 0.02
ConvKB	f(h,r,t)	–	–	–	–	190.30±23.68	0.07±0.01	0.56±0.04	0.88±0.02
	$sim^{e}(h,t)$	251.85±16.28	0.05±0.01	0.43±0.04	0.84±0.01	272.36±19.87	0.04±0.01	0.40±0.05	0.82±0.01
	$sim_{\mathrm{BMM}}^{e}$	376.43±29.23	0.03±0.01	0.29±0.03	0.76±0.02	358.25±35.73	0.03±0.01	0.30±0.04	0.77±0.02
	$sim_{\mathrm{BMA}}^{e}$	312.70±27.68	0.04±0.01	0.38±0.05	0.80±0.02	289.41±31.46	0.04±0.01	0.40±0.04	0.81±0.02
ConvKB-D	f(h,r,t)	–	–	–	–	320.95±59.89	0.06±0.02	0.40±0.07	0.79±0.04
	$sim^{e}(h,t)$	239.33±51.07	0.08±0.01	0.52±0.06	0.85±0.03	230.42±50.91	0.07±0.02	0.51±0.07	0.85±0.03
	$sim_{\mathrm{BMM}}^{e}$	177.11±36.13	0.09±0.02	0.58±0.06	0.89±0.02	160.34±27.41	0.09±0.02	0.64±0.05	0.90±0.02
	$sim_{\mathrm{BMA}}^{e}$	**145.63** ± 32.35	0.11±0.02	**0.66** ± 0.06	**0.91** ± 0.02	**133.70** ± 25.35	0.11±0.02	**0.70** ± 0.05	**0.91** ± 0.02

a Results are reported based on 10 folds. Graph embedding methods are trained using the scoring function f(g,r,d) and evaluated under the scoring functions f(g,r,d), $sim^{e}(g,d)$, $sim_{\mathrm{BMA}}^{e}(P_{g},P_{d})$. Bolded values show the best values in each column. Shaded rows highlight the evaluation under the scoring function $sim_{\mathrm{BMA}}^{e}(P_{g},P_{d})$. ↓ means that lower values are better and ↑ means that higher values are better.

**Figure 2:**
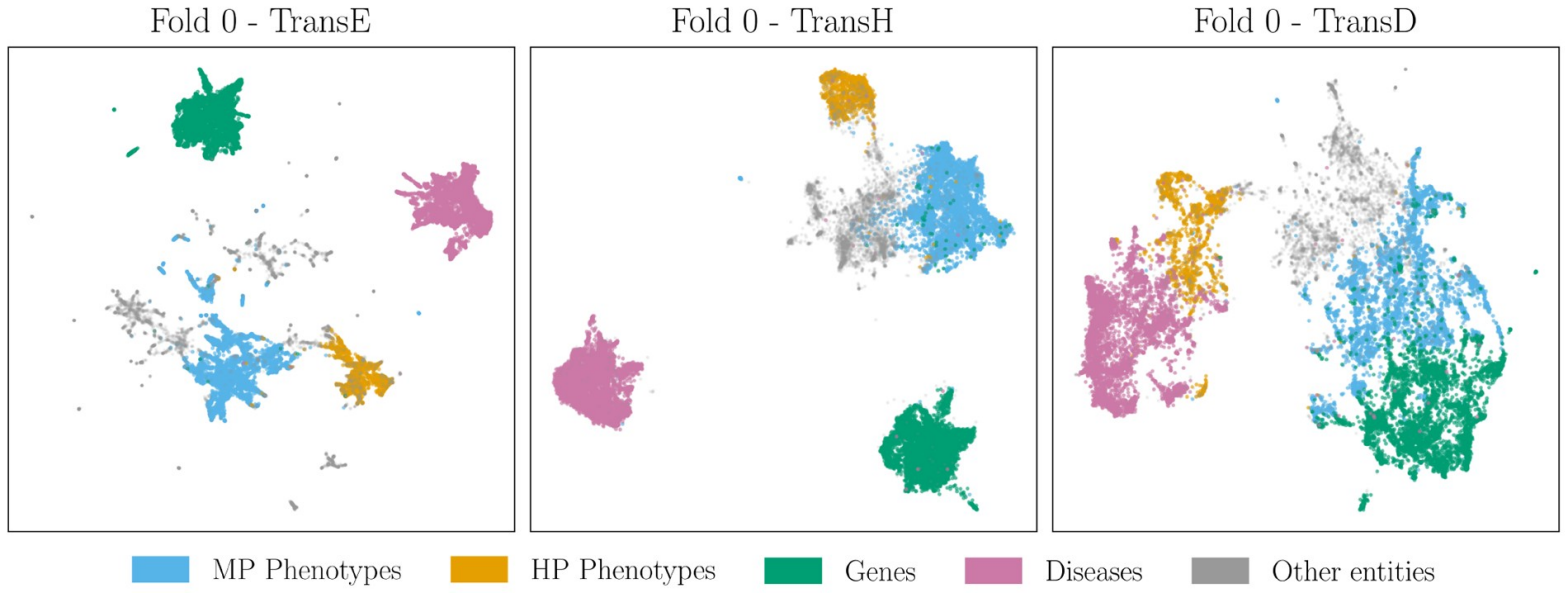
UMAP representation of learned embeddings for methods TransE, TransH, and TransD on the first fold. TransE learns a distance function directly, therefore, the embeddings are scattered across the latent space. TransH and TransD learn a projection function; therefore, the initial latent space captures similarity features of embeddings.

In TransE, embeddings are optimized to predict relations using the distance-based scoring function f(g,r,d) directly. Consequently, evaluation metrics based on f(g,r,d) yield the best results for this model. In contrast, TransH and TransD project entity embeddings into a relation-specific subspace before computing the score. Because the model relies on these projections to handle relational properties, the base embeddings are free to capture intrinsic semantic similarities. This explains why similarity-based metrics ($sim^{e}(g,d)$, $sim_{\mathrm{BMM}}^{e}(P_{g},P_{d})$, $sim_{\mathrm{BMA}}^{e}(P_{g},P_{d})$) outperform the standard scoring function f(g,r,d) for TransH and TransD. Furthermore, TransD outperforms TransH due to its more expressive projection mechanism. Figure 2 illustrates the UMAP (McInnes *et al.* 2018) projections of the learned embeddings. TransE embeddings are widely dispersed across the latent space to satisfy various translational constraints directly. Conversely, TransH and TransD embeddings exhibit distinct clustering; by offloading relational complexity to the projection step, the base embeddings effectively encode semantic similarity.

We also analyse ConvKB, which uses a convolutional neural network on the concatenated embeddings of entities and relations. Originally, ConvKB is initialized with pretrained TransE embeddings, and given that TransD outperforms TransE in our analysis, we initialized ConvKB with TransD embeddings as well, and named it ConvKB-D. ConvKB improves TransE, which shows that the convolutional neural network enhances the similarity features of original TransE embeddings. On the other hand, while ConvKB-D improves TransD under $sim^{e}(g,d)$, its performance under $sim_{\mathrm{BMA}}^{e}(P_{g},P_{d})$ does not change, suggesting that the similarity features learned by TransD cannot be further improved by the convolutional neural network of ConvKB-D.

In both types of measures, semantic similarity measures and embedding similarity, we notice that the aggregation function BMA performs better than BMM; therefore, we rely solely on BMA for the rest of the experiments.

### 3.2 Inductive approach for GDA prediction

In clinical settings, the GDA prediction task frequently involves novel diseases or patients with previously uncharacterized conditions. While the set of genes remains stable, new diseases and phenotypic manifestations continue to be discovered, particularly for rare Mendelian disorders. This presents a fundamental limitation for transductive embedding approaches, which require diseases to be present during the training phase to generate their embeddings.

The key insight of our inductive approach is that, while diseases may be novel, the phenotypes used to describe them are drawn from a stable, predefined ontology. Semantic similarity measures are inherently inductive because they operate on phenotypes rather than directly on diseases or genes. Pairwise similarity between phenotypes is aggregated using an aggregation function such as the BMA. This allows semantic similarity to be applied to any disease characterized by known phenotypes, even if the disease itself was not seen during training.

We extend this intuition to embedding-based approaches by computing BMA scores between phenotype embeddings rather than directly comparing gene and disease embeddings. Given a gene *g* with phenotypes $P_{g}$ and a disease *d* with phenotypes $P_{d}$, we calculate the BMA score based on pairwise similarities between phenotype embeddings using $sim_{\mathrm{BMA}}^{e}(P_{g},P_{d})$. This approach allows us to predict associations for any disease described by a set of phenotypes, even if the disease itself was not included during training.

To evaluate our inductive approach, we conducted experiments using the TransD and ConvKB-D embedding models with the four graph structures of increasing complexity described in Section 2.2 (Fig. 1).

Table 3 presents the performance of our inductive BMA-based approach across these graph structures. For both TransD and ConvKB-D, while Graph 1 and Graph 2 can accumulate more predictions in the top 3, Graph 4 becomes better from top 10 onwards and also in averaged metrics such as MR and AUC. In both methods, for Graph 4, the inductive approach obtains AUC of 0.92, which is comparable to the transductive approach despite the more challenging task of generalizing to unseen diseases, i.e. inductive inference. This demonstrates that learning from phenotype patterns is nearly as effective as directly learning from GDAs. Additionally, the metrics achieved for our methods significantly improve over several standard semantic similarity measures such as SimGIC, Resnik, and Lin with different aggregation methods such as BMA and BMM, confirming that learned phenotype embeddings can capture more complex relationships than handcrafted similarity measures based solely on IC, while still retaining the ability for inductive inference. To demonstrate statistical significance, we conducted a Wilcoxon signed-rank test comparing Lin-BMA (the best baseline) and ConvKB-D G4 across the 10 folds, obtaining a *P*-value of 1.178 × 10^−10^ on the ranks produced by both methods, indicating that the improvement is statistically significant. A per-fold boxplot comparison of Lin-BMA and ConvKB-D G4 is provided in Fig. 1, available as supplementary data at *Bioinformatics* online, showing that ConvKB-D consistently improves over Lin-BMA on MR, Hits@10, Hits@100, and AUC. These results validate that our approach remains effective when applied to diseases not seen during training, making it suitable for real-world clinical applications where newly characterized diseases (characterized as sets of phenotypes) are considered.

**Table 3:** Performance of different graph structures using TransD and ConvKB-D with the BMA-based approach.^a^

Method	**MR (** ↓ **)**	**MRR (** ↑ **)**	**H@1 (** ↑ **)**	**H@10 (** ↑ **)**	**H@100 (** ↑ **)**	**AUC (** ↑ **)**
SimGIC	274.92±31.21	0.08±0.02	0.04±0.02	0.16±0.03	0.50±0.05	0.82±0.02
Resnik-BMM	221.53±26.04	0.08±0.01	0.03±0.01	0.17±0.02	0.51±0.06	0.86±0.02
Resnik-BMA	176.01±18.07	0.12±0.02	0.05±0.02	0.25±0.03	0.62±0.04	0.89±0.01
Lin-BMM	209.27±16.12	0.08±0.01	0.03±0.01	0.16±0.02	0.52±0.03	0.87±0.01
Lin-BMA	164.67±13.68	0.12±0.01	0.05±0.02	0.25±0.03	0.64±0.03	0.89±0.01
TransD G1	144.64±27.26	**0.13** ± 0.01	**0.06** ± 0.01	0.28±0.03	0.68±0.05	0.91±0.02
TransD G2	133.33±19.68	**0.13** ± 0.02	0.05±0.02	0.28±0.03	0.69±0.04	**0.92** ± 0.01
TransD G3	146.96±33.94	0.12±0.02	0.05±0.02	0.27±0.04	0.68±0.06	0.91±0.02
TransD G4	**123.02** ± 27.58	**0.13** ± 0.02	0.05±0.02	**0.29** ± 0.03	**0.73** ± 0.04	**0.92** ± 0.02
ConvKB-D G1	141.59±26.49	**0.13** ± 0.01	**0.06** ± 0.01	**0.29** ± 0.03	0.68±0.05	0.91±0.02
ConvKB-D G2	132.72±20.12	**0.13** ± 0.02	**0.06** ± 0.02	0.27±0.03	0.70±0.04	**0.92** ± 0.01
ConvKB-D G3	148.27±37.66	0.12±0.02	0.05±0.01	0.27±0.04	0.67±0.06	0.91±0.02
ConvKB-D G4	125.68±28.25	0.12±0.02	0.05±0.02	**0.29** ± 0.04	**0.73** ± 0.04	**0.92** ± 0.02

a Results are based on 10-fold cross-validation for Graph 3 and Graph 4, ensuring test diseases were not seen during training. Bolded values indicate best performance for each metric. ↓ means that lower values are better and ↑ means that higher values are better.

## 4 Discussion

### 4.1 Inductive GDA prediction

Inductive approaches for GDA prediction are critical for addressing the challenges of rare genetic disease diagnosis. The majority of Mendelian diseases are rare, with new conditions continually being characterized. Traditional transductive embedding approaches cannot handle previously unseen diseases without complete retraining, severely limiting their clinical utility. Our inductive method addresses this limitation by enabling predictions for novel diseases based solely on their phenotypic profiles.

This capability is particularly important when integrating GDA prediction into variant prioritization (Althagafi *et al.* 2024). These systems combine phenotype-based gene prioritization with variant pathogenicity metrics to identify causative variants in patients with genetic disorders. By adopting our inductive approach, these systems can handle patients with previously uncharacterized diseases or novel combinations of phenotypes without requiring prior knowledge of specific disease entities during model training.

Our approach extends the state-of-the-art in multiple ways. First, unlike traditional semantic similarity measures like Resnik or Lin, which rely on handcrafted metrics, our method leverages knowledge graph embeddings to learn relationships between phenotypes across species. Second, unlike previous embedding approaches that require diseases to be present during training, our method operates at the phenotype level, making it inherently inductive. Third, we retain the benefits of supervised learning by incorporating GDAs during training while maintaining the ability to generalize to unseen diseases. This balance between supervision and induction is likely the most significant advancement over existing approaches, and may open up possibilities for future improvements in predicting GDAs.

### 4.2 Embedding model performance

Our experimental results demonstrate that TransD consistently outperforms other embedding models such as TransE and TransH for GDA prediction. TransD’s scoring function mechanism maps entities into entity-relation-specific spaces and computes scores f(h,r,t) in the target space. Thus, in the origin space, base embeddings obtain similarity features that are then projected to similar regions in the target space. This property of TransD makes it well-suited for capturing the relationships in our graph. Additionally, training a ConvKB model with TransD features can slightly improve the performance.

While the UPheno ontology alone (Graph 1) provides a foundation for phenotype comparison, the addition of gene–phenotype associations (Graph 2), disease–phenotype associations (Graph 3), and known GDAs (Graph 4) each contribute to improved predictive performance.

We observed that performance decreases when moving from Graph 2 to Graph 3 (adding disease–phenotypes without gene–disease links) for projection-based methods like TransD. We attribute this to the structural differences between gene and disease annotations. Genes are annotated with the MP ontology (MP) while diseases use the HPO; although linked via UPheno, the phenotype annotations have different annotation frequencies, granularities, and biases.

In the absence of direct gene–disease links, embedding methods like TransD, which project entities into relation-specific spaces, will segregate genes and diseases into distinct regions of the latent space based on these annotation differences. The GDAs in Graph 4 then act as an alignment signal, forcing the model to bridge this domain gap and map genes and diseases into a shared manifold.

Conversely, we observe that TransE performance degrades with this supervised signal (Table 2). TransE models the associated_with relation as a translation vector *r*, enforcing the constraint h+r≈t. This translation explicitly separates the gene and disease embeddings by the magnitude of *r* in the vector space. Because our inference approach relies on direct embedding similarity (where higher similarity corresponds to closer proximity), this forced separation makes the phenotypes associated with genes and diseases more dissimilar in the latent space, thereby degrading the performance of similarity-based ranking.

Overall, our findings demonstrate that each layer of biological knowledge can add a signal that the embedding models may leverage. We also note that Graph 2 contains the information that can be used by the semantic similarity measures, as IC is computed over genes; Graphs 3 and 4 are able to utilize more information than available to semantic similarity measures.

Notably, the supervised model (Graph 4) achieves substantially higher performance than unsupervised alternatives, highlighting the importance of known GDAs as a supervision signal. The need for a supervised signal has also been shown in all prior studies that rely on embeddings for computing GDAs. However, unlike previous supervised embedding approaches (Alshahrani and Hoehndorf 2018, Smaili *et al.* 2018, 2019, Chen *et al.* 2021a,b) that sacrifice inductive capabilities, our method maintains the ability to generalize to unseen diseases through the BMA-based phenotype comparison.

### 4.3 Comparison with semantic similarity measures

Our comparison with semantic similarity measures reveals important insights about the strengths of embedding-based approaches. While semantic similarity measures have been widely used due to their simplicity and interpretability, they primarily rely on the IC of the most informative common ancestor in the ontology hierarchy. This approach neglects more complex relationships and cannot adapt to the specific patterns in GDA data.

In contrast, our embedding-based approach learns latent representations that capture both hierarchical and nonhierarchical relationships in the ontology. The TransD model can identify axiom patterns in how phenotypes relate to each other across species, which may explain its superior performance. Additionally, by incorporating a supervised signal during training, our model can learn which phenotype patterns are most relevant for predicting GDAs, rather than relying solely on general semantic similarity.

### 4.4 Limitations

Our approach has several limitations. First, INDIGENA requires that all query phenotypes are already represented in the UPheno ontology; truly novel phenotypes that have not yet been added to the ontology cannot be handled. In practice, this means that the inductive capability of our method applies to novel *combinations* of known phenotypes (i.e. novel diseases or patients), but not to entirely new phenotype terms. Second, our evaluation is restricted to the mouse-to-human transfer setting using MGI and OMIM data. While this is a well-established benchmark for GDA prediction, the generalizability of our results to other model organisms or phenotype sources remains to be evaluated. Third, we do not compare against graph neural network-based inductive methods such as GraphSAGE (Hamilton *et al.* 2017), which represent the standard inductive baselines in the graph machine learning literature. Such methods could potentially learn inductive representations directly, although they would require adaptation to handle the ontology structure and the set-based nature of phenotype profiles. Finally, the choice of the BMA aggregation function, while well-motivated by prior work in semantic similarity, is fixed; learning the aggregation function jointly with the embeddings could further improve performance.

## 5 Conclusions

Our study demonstrates that inductive, supervised GDA prediction can successfully address the limitations of traditional approaches. By operating at the phenotype level rather than directly at the disease level, our method can generalize to novel diseases based solely on their phenotypic manifestations. This capability is particularly valuable for rare disease diagnosis where previously uncharacterized conditions regularly emerge.

The framework we have developed has implications for clinical genomics. By enabling accurate prediction of GDAs for novel diseases, our approach can improve the prioritization of candidate genes in diagnostic settings. This can potentially reduce the time and cost of rare disease diagnosis, ultimately leading to earlier and more effective treatment interventions.

## Supplementary Material

btag325_Supplementary_Data

## Data Availability

The data underlying this article are available in the article and in its online supplementary material.
