# Supplementary material for: INDIGENA: inductive prediction of disease–gene associations using phenotype ontologies
Source: Bioinformatics. 2026 May 21;42(6):btag325. doi: 10.1093/bioinformatics/btag325 (PMC13242216; doi:10.1093/bioinformatics/btag325)
Supplement: btag325_Supplementary_Data [file btag325_supplementary_data.pdf]

INDIGENA: inductive prediction of disease–gene associations using phenotype ontologies
Supplementary Material

Fernando Zhapa-Camacho Robert Hoehndorf

Table 1: Hyperparameters

Model	Embedding dim	Batch size	Learning rate	Num filters
Transductive G3				
TransE	100	8192	0.001	
TransH	200	2048	0.001	
TransD	400	2048	0.001	
ConvKB	100	8192	0.0001	100
ConvKB-D	100	8192	0.0001	100
Transductive G4				
TransE	100	2048	0.001	
TransH	200	1024	0.001	
TransD	100	2048	0.001	
ConvKB	100	8192	0.0001	100
ConvKB-D	100	8192	0.00001	200
Inductive G1				
TransD	400	8192	0.001	
ConvKB-D	100	8192	0.0001	100
Inductive G2				
TransD	400	4096	0.001	
ConvKB-D	100	4096	0.0001	100
Inductive G3				
TransD	400	8192	0.001	
ConvKB-D	100	8192	0.0001	100
Inductive G4				
TransD	400	8192	0.001	
ConvKB-D	100	2048	0.0001	200

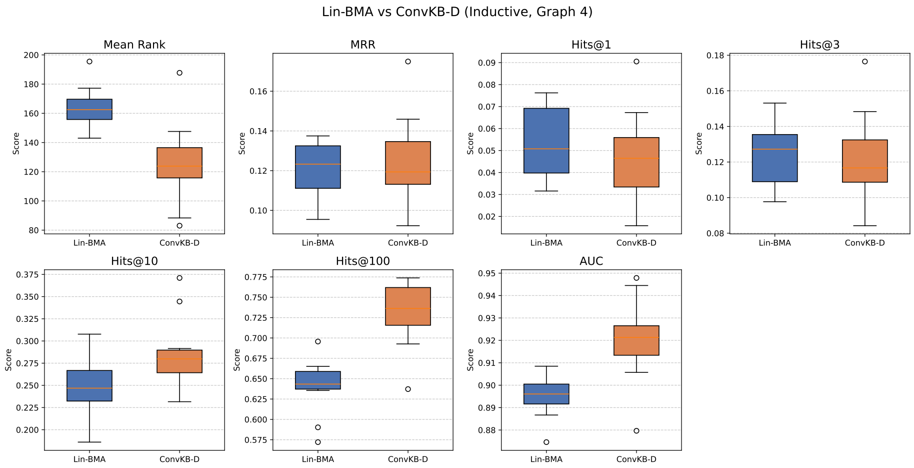

Figure 1: Boxplot comparison of Lin-BMA and ConvKB-D in the inductive setting on Graph 4 across 10 folds. ConvKB-D outperforms Lin-BMA on Mean Rank, Hits@10, Hits@100, and AUC, while both methods show comparable performance on Hits@1, Hits@3, and MRR.
